# Electromagnetic tool for the endoscopic creation of colon anastomoses—development and feasibility assessment of a novel anastomosis compression implant approach

**DOI:** 10.1007/s11548-022-02722-z

**Published:** 2022-09-10

**Authors:** Jana Steger, Anne Zimmermann, Thomas Wittenberg, Petra Mela, Dirk Wilhelm

**Affiliations:** 1 Research Group Minimally-Invasive Interdisciplinary Therapeutical Intervention (MITI), Klinikum Rechts Der Isar, Technical University of Munich, Trogerstraße 26, 81675 Munich, Germany; 2 Department of Computer Science, Institute of Visual Computing, Friedrich-Alexander-University Erlangen-Nuremberg, Erlangen, Germany; 3 Department of Mechanical Engineering and Munich Institute of Biomedical Engineering, Chair of Medical Materials and Implants, TUM School of Engineering and Design, Technical University of Munich, Garching, Germany; 4 Klinikum Rechts Der Isar, TUM School of Medicine, Clinic and Policlinic for Surgery, Technical University of Munich, Munich, Germany

**Keywords:** Endoscopic, Surgery, Anastomosis, Electromagnet, Implant

## Abstract

**Background:**

Colorectal anastomoses are among the most commonly performed interventions in abdominal surgery, while associated patient trauma is still high. Most recent trends of endoscopic anastomosis devices integrate magnetic components to overcome the challenges of minimally invasive surgery. However, the mutual attraction between magnetic implant halves may increase the risk of inadvertently pinching healthy structures. Thus, we present a novel anastomosis device to improve system controllability and flexibility.

**Methods:**

A magnetic implant and an applicator with electromagnetic control units were developed. The interaction of magnetic implants with the electromagnets bears particular challenges with respect to the force-related dimensioning. Here, attraction forces must be overcome by the electromagnet actuation to detach the implant, while the attraction force between the implant halves must be sufficient to ensure a stable connection. Thus, respective forces were measured and the detachment process was reproducibly investigated. Patient hazards, associated with resistance-related heating of the coils were investigated.

**Results:**

Anastomosis formation was reproducibly successful for an implant, with an attraction force of 1.53 $\pm 0.3 N$, resulting in a compression pressure of $0.0048 \frac{N}{{\mathrm{mm}}^{2}}$. The implant was reproducibly detachable from the applicator at the anastomosis site. Coils heated up to a maximum temperature of ${T}_{\mathrm{max}}=41.6 \pm 0.1^\circ \mathrm{C}$. Furthermore, we were able to establish a neat reconnection of intestinal bowel endings using our implant.

**Discussion:**

As we achieved nearly equal compression forces with our implant as other magnetic anastomosis systems did (Magnamosis™: 1.48 N), we concluded that our approach provides sufficient holding strength to counteract the forces acting immediately postoperatively, which would eventually lead to an undesired slipping of the implant halves during the healing phase. Based on heat transfer investigations, preventive design specifications were derived, revealing that the wall thickness of a polymeric isolation is determined rather by stability considerations, than by heat shielding requirements.

## Introduction

Colorectal resections are among the most commonly performed interventions in abdominal surgery. In 2008, there were approximately 2.1 million new diagnoses of colorectal carcinoma all over the world [1], with a high percentage of them requiring surgical intervention. Additionally, only in the US, due to diverticular disease, more than 22.000 surgical resections are conducted every year, as for 2005 [2]. Adding the various other indications, we must assume that more than 1 million individuals worldwide undergo colon resection every year. After the bowel segment has been removed, the remaining colonic endings must be connected to reestablish the continuity of the digestive tract. For large parts of the colon, forming the so-called “anastomosis” is the most invasive step in the procedure and the associated intraoperative patient trauma is still high.

One important step to allow a less invasive treatment and a decrease in patient burden, is to minimize the access trauma. Therefore, devices are required, that assist the surgeon and allow to overcome the challenges of complex minimally invasive surgery, which are in the first line restricted manipulation space, indirect instrument handling capabilities and lack of adequate force application. To overcome these critical issues, magnets with their inherent functionality of mutual attraction are an increasingly recognized tool for laparoscopic and endoscopic instruments and interventions [3].

Following this trend, latest approaches in anastomosis research are based on two-part magnetic implants, designed to enable endoscopic formation of the reconnection. These systems apply an enduring pressure on the tissue in the compression zone. While it grows together in one area of the joined intestinal endings, tissue becomes necrotic in the center of the lumen. In this way, the compression implant is excreted together with the necrotic segment via natural stool passage [4].

Promising results for the anastomosis formation via magnetic implants have been achieved in first studies with the Magnamosis™ system (Magnamosis™, Inc., San Francisco, CA) [5–10] and the Incisionless Magnetic Anastomosis System (IMAS, GI Windows, West Bridgewater, MA, USA) [11, 12]. However, laparoscopic support was still needed for coupling assistance [11], and with respect to endoscopic navigation and implant application, it must be considered that the mutual attraction between magnetic implant halves may increase the risk of positioning failure, and thus, of inadvertently pinching healthy structures. This is especially problematic, as due to the endoscopic application, the access possibilities for the surgeon are very limited and readjustment by hand is not possible.

The goal of the herein presented research was to develop a novel anastomosis instrument that allows for maximal system controllability and flexibility with respect to implant de- and reattachment, to facilitate the establishment of an end-to-end anastomosis, as this configuration type comes closest to the native bowel functionality and allows to save time and material. Therefore, a magnetic implant and an application device with electromagnetic control units to flexibly pick up, deploy and join an anastomosis implant was developed.

## State of the art

The use of electromagnets in surgical interventions has found more and more diverse application in recent years. For example electromagnets have been used extracorporeally, to achieve proper tissue counter-traction for the en bloc resection of early tumors in the digestive tract during endoscopic submucosal dissection. Tissue traction during endoscopic procedures is especially challenging due to the limitations posed by using only a single instrument. Therefore, Kobayashi et al. developed, performed and published the Magnetic-Anchor-Guided ESD (MAG-ESD) procedure. The intervention involves an endoscopically positioned magnet, which is fixed to the gastric mucosa, and an extracorporeally controlled electromagnet that manipulates the intracorporeally positioned magnet. By moving the electromagnet, the gastric mucosa can be dragged to neatly expose the targeted tissue area, where the dissection is supposed to be performed [3, 13].

Another field of application for externally used electromagnets in gastrointestinal surgery are actively controlled capsule endoscopes. Capsule endoscopes are pills, comprising an optical system, to monitor the lumen of the stomach, intestine or colon. The integration of an active locomotion mechanism is required to enable not only diagnostic, but also therapeutic procedures. One approach proposed is the manipulation of intracorporeally located magnetic capsules with external electromagnets [14]. This is supposed to improve control and modulation of the forces acting on the endoscopic capsule compared to permanent magnets.

However, to create sufficiently large magnetic fields also in deeply seated regions of the human body, large electromagnetic units with extensive spatial demand are required. For example, even micro-surgery applications, with workspaces of only a few millimeters, afford external electromagnets in highly over-scaled dimensions which are not feasible for an operating room setting. Sikoriski et al. [15] therefore proposed a new micro-robotic device, using three miniaturized electromagnets included in a flexible catheter, to deliver and control micro-agents, directly at the surgical site. They postulate that the integration of magnetic actuation systems on catheters, endoscopes and needles has high potential, providing well-defined and stable workspace [15].

Another most recent example for the use of electromagnets within the human organism is variable stiffness manipulators (i.e., for flexible endoscopy), which are based on magneto-rheological compounds (MRC). Rheological properties can be tuned by the variation of an externally applied magnetic field. The manipulator’s shaft consists of MRC-based rings, of which each is boxed in between two electromagnets, generating the magnetic field. Between every two adjacent electromagnets, there is a non-magnetic spacer, which prevents the magnetic fields from leaking between the joints. Four wires are used for the manipulator’s tip movement. The MRC joints behave differently depending on the magnetic field, they are exposed to. The MRC’s stiffness increases, if it is exposed to an attractive magnetic field, and it decreases in the presence of non-magnetic fields, when there is no current applied to the electromagnets. Only the latter state allows the joints to bend if one of the four wires is actuated [16].

In this contribution, we present a novel endoscopic, end-to-end anastomosis (EEA) device, which combines the use of a magnetic anastomosis implant with the approach of integrating electromagnetic control units into a manipulator that is designed to flexibly pick up, deploy and join the implant halves. The suitability of the approach was evaluated in terms of achievable compression forces and the electromagnet power, which determines implant handling and detachment capabilities. Furthermore, potential patient hazards were assessed with respect to electromagnetic heating effects.

## General concept

**Fig. 1:**
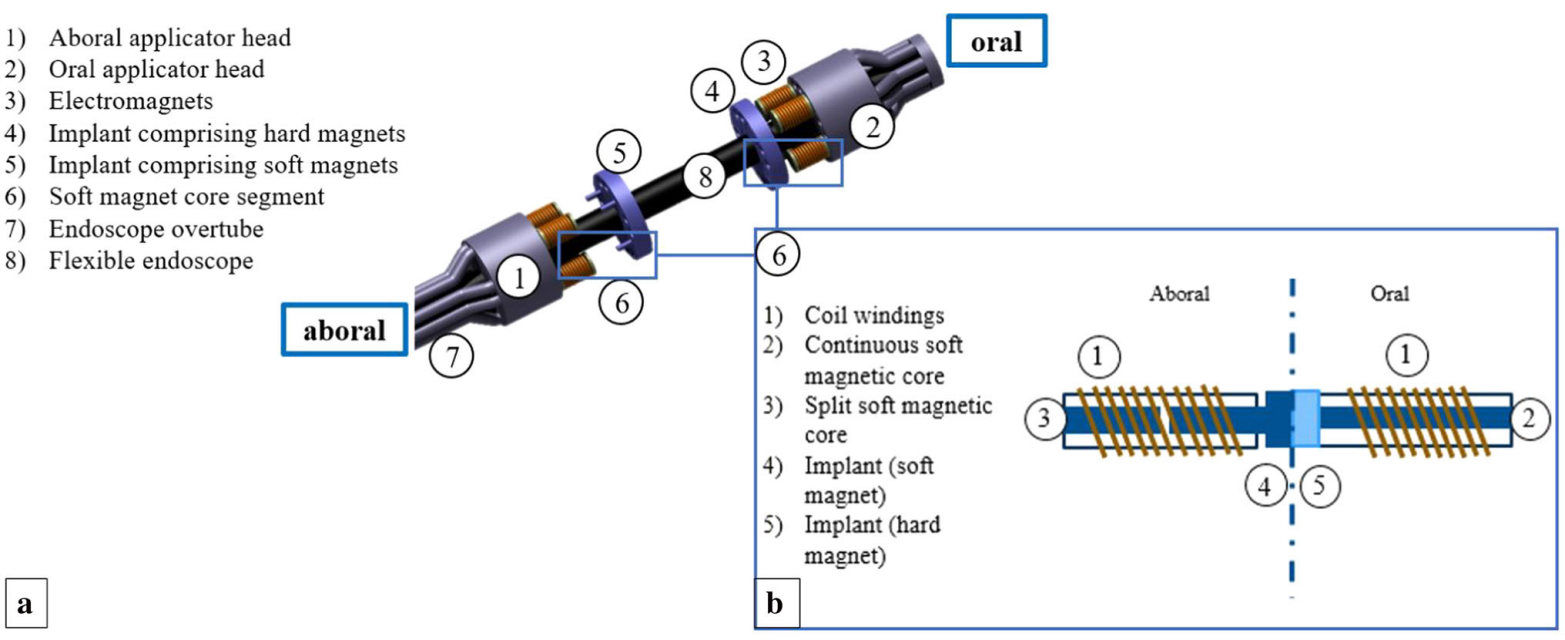
**a** Schematics of the anastomosis device comprising a two part applicator (1, 2), an implant (4, 5) and an endoscope overtube (7). Electromagnets (3) are used for control of implant de- and attachment from/to applicator heads. A flexible endoscope is used as carrier unit (8) for the system. At the aboral side, a soft magnet segment protrudes from the implant, into the electromagnetic coil (6). **b** Detailed schematics of the electromagnet mechanisms and implant units on both sides. Coil windings (1), continuous soft magnetic core (2), split soft magnetic core (3), implant comprising soft magnets (4), implant comprising hard magnets (5). Components positioned at the endoscope tip, i.e., closer to the patient’s mouth, are described by the term “oral” and system units positioned at the endoscope shaft, i.e., toward the patient’s anus, are described by the term “aboral”

The developed system consists of an applicator and a compression implant, each comprising two units. It follows an over-the-tube design, which can be mounted on conventional flexible endoscopes. This allows to avoid integration of high-priced optical modules and electronic components, in order to enable visualization of the navigation path and intervention site in a cost-effective way. To manipulate bowel endings independently of each other and adjust colon edges, relative movement between oral and aboral applicator heads is enabled. (The terms “oral” and “aboral” are used in the following as positioning references for the anastomosis device components. “Oral” describes the units positioned at the endoscope tip, i.e., closer to the patient’s mouth, and “aboral” describes the system units positioned at the endoscope shaft, i.e., closer to the patient’s anus.) (see Fig. 1)

While the aboral applicator entity is connected to the handling unit, the oral one is attached to the endoscope shaft. Both applicator units integrate four electromagnetic coils, carrying one implant half. Each of these implant halves includes four magnetic elements (oral: hard/permanent magnets, aboral: soft magnets). In the oral applicator unit, the electromagnetic coils include a soft magnetic, monolithic core, while in the aboral applicator head, the cores are split in two parts. One of these two portions is anchored in the coil, and the other one protrudes from the implant into the electromagnet (system schematic Fig. 1). This hull like structure supports the reliable attachment of the implant to the applicator during navigation through the colon.

**Fig. 2:**
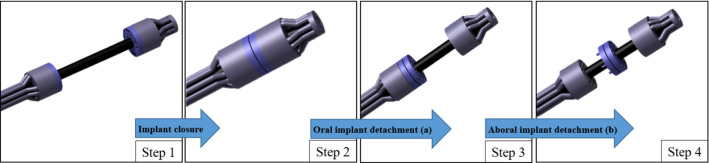
Schematics of the surgical procedure. Step 1: Starting configuration and positioning in the oral and aboral lumen. Step 2: Implant closure to achieve bowel reconnection; Step 3: Repulsion of permanent magnetic segments in the oral implant by establishment of a repulsive magnetic field (electromagnets); Step 4: Rejection of soft magnets in the aboral implant by establishment of a repulsive magnetic field (electromagnets)

For the creation of an anastomosis, the oral and aboral applicator heads are positioned in the center of the bowel lumina (Fig. 2 Step 1). The colon margins are draped around the implant halves, into the compression zone, and the endings are approximated by pushing/pulling the endoscope and/or the overtube to move the heads separately. Once the joining interface of the tissue layers in between the implant halves is deemed to be continuous, the implant can be closed (Fig. 2 Step 2). Joining the implant halves, the hard magnets of the oral side magnetize the soft magnetic elements of the aboral side, whereby the tissue is subjected to a permanent pressure. Subsequently, the oral implant half is detached from the applicator head (Fig. 2 Step 3) by a current pulse through the electromagnets and the resulting repellent magnetic force. Finally, the closed implant is repelled from the aboral applicator head. The application device is pulled out of the colon, while the anastomosis implant stabilizes the intestinal reconnection until the implant is excreted.

## Material and methods

**Fig. 3:**
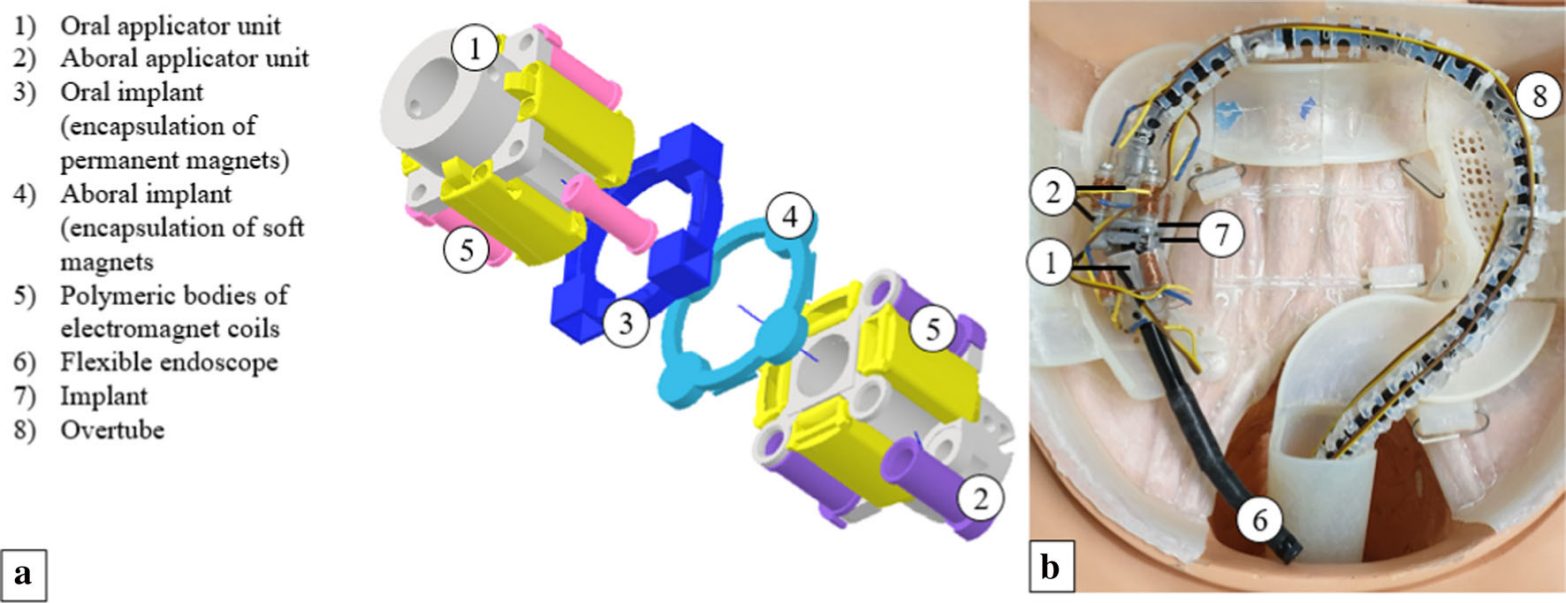
Schematics of the application device and implant units. Numbers indicate the most important parts, described on the side bar of (**a**) Applicator prototype mounted on a flexible endoscope within a mechanical trainer

**Fig. 4:**
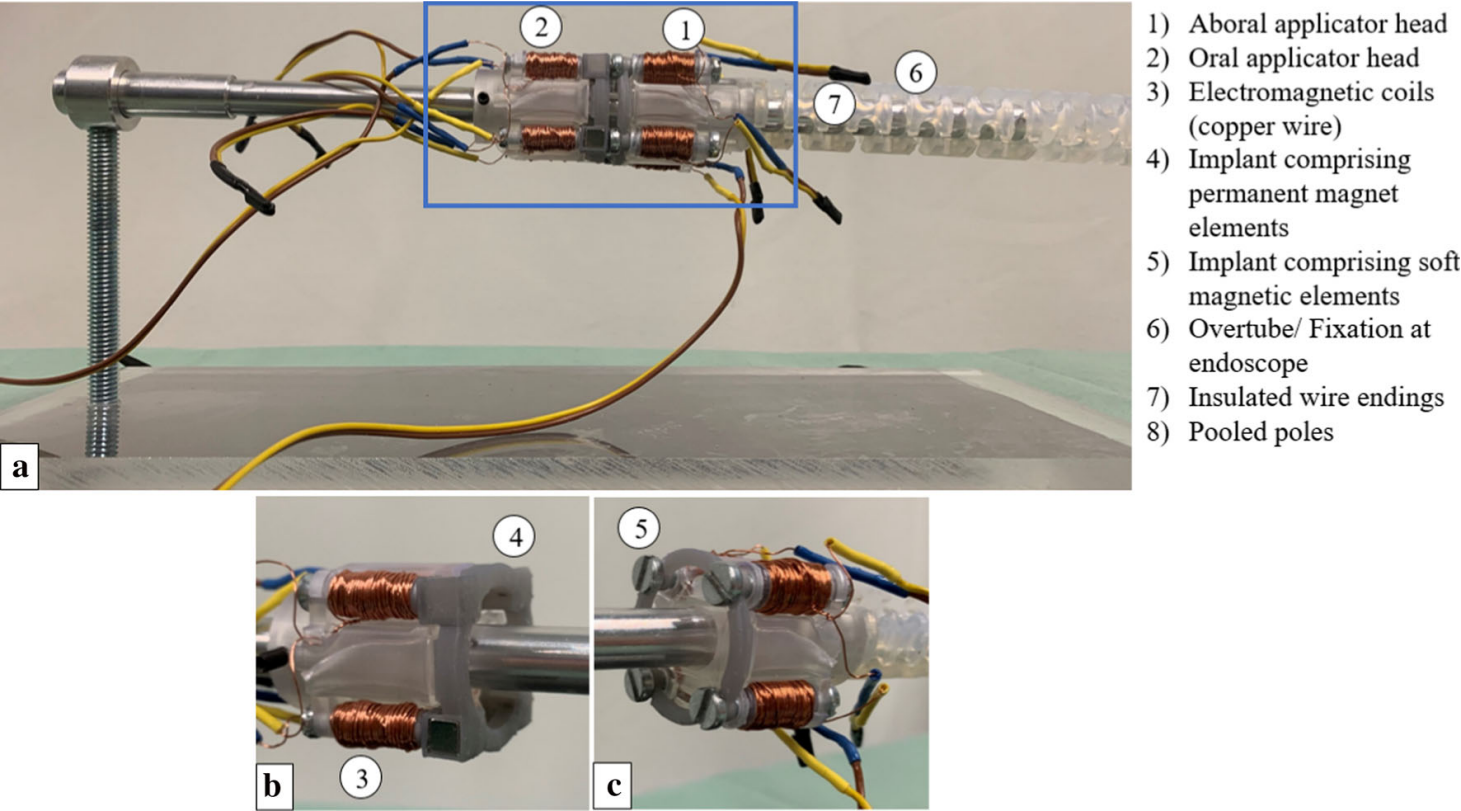
**a** Applicator and implant prototype mounted on the experimental setup **b** Oral applicator and implant unit (permanent magnets) **c** Aboral applicator and implant unit (soft magnets)

To assess the feasibility of the concept in terms of achievable compression forces, the electromagnet power, and potential patient hazards with respect to electromagnetic heating effects, a prototype, comprising implant and applicator, was built (see Figs. 3 and 4), and respective experiments were conducted. There was no IRB approval obtained and no written consent defined, as this was not required for the present study design.

### Prototype description

The prototype consists of two implant halves, two applicator units (oral/ aboral) and eight polymeric coil bodies (four per applicator unit). The implant halves and coil bodies are manufactured with the Formlabs2 stereolithographic process (Formlabs GmbH, Berlin, Germany) from the material “Tough 1500.” This resin is used for the filigree components of the prototype, due to its lower tensile modulus compared to standard resins, and therefore higher flexibility and decreased risk of breakage [17]. The implant carrier units are printed with the Formlabs3B (Formlabs GmbH, Berlin, Germany), using “Clear standard resin,” which is a material designated for components affected by low mechanical loads, but high accuracy requirements [18].

For the oral implant, four equal cubic N52 magnetized neodym magnets^1^ (EarthMag GmbH, Dortmund, Germany) with a side length of 5 mm are used. The permanent magnets are encapsulated by a polymeric sheath of 0.6 mm wall width. The oral implant includes soft magnetic mild steel screws^2^ with a standard M4 thread, which protrudes into the electromagnetic coils of the applicator when the implant is mounted to support the reliable attachment of the implant to the applicator during navigation through the colon.

For the electromagnetic cores, soft magnetic mild steel screws^2^ with a standard M3 [19] thread in the oral applicator and standard M4 thread in the aboral applicator (magnetic permeability range ${{\mu }_{r}}_{\mathrm{min}}=100; {{\mu }_{r}}_{\mathrm{max}}=800-2000$) [20] are used. Identical M4 screws are utilized within the implant. The “core length ratio” designates the proportion of implant protrusion length to electromagnetic core length, which influences the electromagnet field strength and thus the application device power. In the presented prototype, an implant with a core length ratio of “1/3” (thus $\sim$ 9 mm) and an electromagnetic coil core of 2/3 (thus $\sim 27 mm)$ are used (all ratios given with respect to the total length of a M4 screw).

The polymeric coil bodies of the oral and the aboral implant carrier units have a maximum length of ${l}_{\mathrm{coil}}$=15 mm (winding length of 13 mm). The diameter of the oral polymeric coil bodies is ${\varnothing }_{\mathrm{oral}}^{\mathrm{coil}}$ = 4.6 mm and of the aboral coils ${\varnothing }_{\mathrm{aboral}}^{\mathrm{coil}}$ = 6.4 mm*.* In the aboral applicator head, the coil bodies’ inner diameter is dimensioned 0.8 mm larger than the screw thread diameter, to allow sufficient slack between polymer wall and screw, to avoid mechanical interference (such as tilting, friction, etc.) hampering implant expulsion. The protruding implant section and the electromagnet cores touch each other within the coils. All coils are spooled clockwise from bottom to top with an enameled, 0.3 mm diameter copper wire (Conrad Electronic SE, Hirschau, Germany). The coils comprise 100 windings and are operated at 24 V direct current (3 A). All system dimensions are indicated in Table 1.

**Table 1:** Overview of implant and applicator dimensions

Description	Formula	Magnitude
Outer diameter of oral implant (including permanent magnets)	${\varnothing }_{\mathrm{out}}^{\mathrm{oral implant}}$	35 mm
Outer diameter of the aboral implant (including screw heads)	${\varnothing }_{\mathrm{out}}^{\mathrm{aboral implant}}$	34 mm
Inner diameter of the oral implant	${\varnothing }_{\mathrm{inner}}^{\mathrm{oral implant}}$	22 mm
Inner diameter of the aboral implant	${\varnothing }_{\mathrm{inner}}^{\mathrm{oral implant}}$	24 mm
Outer diameter of the oral applicator unit	${\varnothing }_{\mathrm{out}}^{\mathrm{oral applicator}}$	33.3 mm
Outer diameter of the aboral applicator unit	${\varnothing }_{\mathrm{out}}^{\mathrm{aboral applicator}}$	35.6 mm
Length of oral applicator unit	${l}_{\mathrm{oral}}^{\mathrm{applicator}}$	28 mm
Length of the aboral applicator unit	${l}_{aboral}^{applicator}$	32 mm

The coils of each of the two applicator heads are connected in series. A control unit was designed and, with the help of an ${\mathrm{Arduino}}^{\mathrm{TM}}$ microcontroller board circuit (Arduino™ Uno SMD R3, Italy) and a laboratory power supply (HM305, Hanmatek, Shenzhen, China) unit, the electromagnetic coils of the oral and aboral applicator head can be switched on separately for accurately adjustable periods. Furthermore, the current flow can be inverted for the aboral side, attracting or repelling the respective implant half. The prototype of the entire system is shown in Fig. 4.

### Experiments

#### Implant detachment

The interaction of magnetic implants with the electromagnets bears particular challenges with respect to the force-related dimensioning of individual units. Here, attraction forces must be overcome by the electromagnet operation, to detach the implant. At the same time, the attraction force between the implant halves, which decreases significantly with increasing distance between magnetic partners, must be sufficient to ensure a stable connection and withstand immediately postoperatively occurring forces.

Furthermore, all components must meet the highly restricted space requirements of minimally invasive instruments resulting from narrow access routes. Thus, two kinds of experiment were conducted. The force required to separate magnetic elements of the implant halves was determined as a measure of attraction force. Then, the implant halves were detached from the applicator prototype with integrated electromagnets. Finally, patient hazards associated with intracorporeal use of electromagnets were investigated by measuring resistance-related heating of the coils.

All experiments were performed with porcine colon, obtained from the slaughterhouse on the morning of the respective experimental day. Fatty tissue was removed from the intestine, which was then cut into segments of approximately 6 cm in length. Tissue pieces were paired, and the thickness of two layers was measured in uncompressed state, to determine the amount of tissue clamped in the compression zone.

**Fig. 5:**
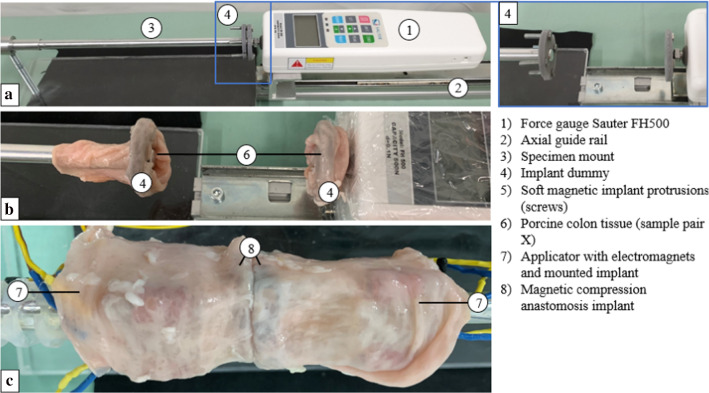
**a** Experimental setup to measure forces acting between implant halves using a measurement force gauge (1), fixed on a slide to axially move it along a force rail (2). Implant dummies (4) were mounted on a metal bar (3) and fixed to the force gauge. **b** Each sample pair (6) is mounted on the implant dummy (4), to measure the adhesion force with respect to the tissue pair thickness. **c** In the second step, the sample pair is mounted on the applicator (7) to test the feasibility of the magnetic implant (8) detachment mechanism

The experimental setup comprised a force gauge (FH500, Sauter, Kern & Sohn GmbH, Germany) (Fig. 5a (1)), fixed on an axially movable slide (Fig. 5a (2)), a stationary bar (specimen mount, simulating the colonoscope) (Fig. 5a (3)) and a two-part implant dummy (Fig. 5a (4)). The implant halves were made of polymer discs, including four neodym cubes, and four soft magnetic counterparts (Fig. 5a (4)). These tissue compression determining elements were identical to those used in the implant (Fig. 4b: permanent magnets, Fig. 4c: soft magnetic screws). One implant dummy half was screwed to the stationary bar and the other one fixed to the force measurement unit (Fig. 5a. The force gauge was pushed towards or pulled away from the opposingly mounted implant dummy half (in a reproducible trajectory to assure a consistent force vector).

The magnetic adhesion force between the implant dummies was measured by axially retracting the force gauge, with two pieces of intestinal tissue placed in the compression zone (Fig. 5b). To account for the colon’s viscoelastic tissue behavior [21] (and associated effects such as creep) under a constant compression pressure, we waited one minute before starting the experiments. The magnetic attraction forces were measured *n* = 5 times per tissue pair by pulling the implant halves apart and the mean value was calculated for each sample pair. In total the separation forces for *n* = 5 test cycles were investigated.

The sample pairs were then each placed on the oral an aboral applicator heads with mounted implants (Fig. 5c), which were joined to close the anastomosis. Subsequently, the implant halves’ were detached from the applicator heads, by actuating the oral and aboral electromagnets consecutively.

#### Electromagnetic heating

Even though electromagnets are an increasingly recognized tool in visceral surgery, their integration in minimally invasive instruments is still considered critical, especially for intracorporeal use. Concern has to be given in respect to electric and electromagnetic compatibility with surrounding tissue and in regard to a rise in temperature, resulting from resistance-based heating of the coils [22, 23]. Thus, experiments were conducted for the typical operation mode of implant detachment, which comprised short repellent impulses. The coils were powered six times for one second each, by automated switch on and off processes, in order to investigate how the system behaves if the implant is repeatedly picked up and disconnected again. A three-second pause was maintained between each pulse. The initial (${T}_{\mathrm{init}}$) and the maximum temperatures (${T}_{\mathrm{max}}$) were recorded, as well as after each individual impulse X (${T}_{x\in [0;7]}$). The experiment was performed with an initial coil temperature equal to room (${T}_{\mathrm{init}}={T}_{\mathrm{room}}$ =$\sim$ 23 °C) or body temperature (about ${T}_{\mathrm{init}}={T}_{\mathrm{body}}$ =$\sim$ 37 °C). Both sets of experiments were repeated *n* = 5 times.

Temperature measurements were taken only on the electromagnets of the aboral side, due to their higher resistance (larger coil $\to$ longer wire) and therefore more critical heating characteristics. For this means, the tip of a thermometer (CP011A, Habor, Shenzhen Xintuo Supply Chain Ltd., Shenzhen, China), with a measuring range between -50 °C and 300 °C and an accuracy of 0.1 °C, was brought into contact with the shell surface of one of the electromagnets. The position was fixed using a tape (Durapore™, 3 M™, Saint Paul, USA) to ensure permanent contact between the prototype and the thermometer throughout the entire experiment duration.

Between all test sets, it was ensured that the coils cooled down until they reached to their respective initial temperature (${T}_{\mathrm{room}}$ or ${T}_{\mathrm{body}}$) to provide equal and comparable preconditions.

## Results

### Implant detachment

We evaluated the compression forces between the implant halves (Figure 5) (using implant dummies) with two colon tissue layers compressed in the gap. Based on this, the compression pressure acting on the tissue was determined. A mean separation force of ${F}_{{\text{electromagnet implant}}}^{\text{separation}}=$ 1.53 N$\pm 0.3$ was measured. This value resulted from averaging the mean value of all measurements per sample pair, over all five test cycles performed. The mean sample tissue thickness was 2.9$\pm 0.3 \mathrm{mm}$.

The pressure active area of the implant herein presented resulted from the four cubic neodym magnets, with a side length of 5 mm each.

$${A}_{\text{electromagnet implant}}=4*{\left(5 {\text{mm}}\right)}^{2}*\pi = 100*\pi =314.16 {\text{mm}}^{2}$$

$${P}_{\text{electromagnet implant}} =\frac{{F}_{{\text{electromagnet implant}}}^{\text{separation}}}{{A}_{{\text{electromagnet implant}}}}=\frac{1.53 N }{314.16 {\text{mm}}^{2} }=0.0048 \frac{N}{{\text{mm}}^{2}}$$

Furthermore, we investigated the implant detachment from the applicator, to assess the applicator’s electromagnetic power. The oral implant half was decoupled from the oral applicator by means of a pulsed current, resulting in a resistance-induced voltage drop (to 11 V) during operation. In the next step, the aboral implant half was ejected from the aboral applicator head, leading to a voltage drop to 14 V. Detachment of the closed implant from the oral and the aboral applicator units, thus repelling the magnetic elements by means of the electromagnets, was reproducibly successful.

### Temperature development for pulsed coil powering

In the second part of experiments, concern was given to the temperature rise, resulting from resistance-based heating of the coils.

Temperature recording time step *x* = 0 corresponded to the initial coil temperature ($${T}_{0}{=T}_{init}$$), which was either room ($${T}_{\mathrm{room}}^{1s}=$$ 22.$$9\pm $$ 0.5 °C) or body temperature $$({T}_{\mathrm{body}}^{1s}=$$ 37.0 °C$$\pm 0.0$$). Temperature measurements ($${T}_{x}^{1 s}$$) were repeated every 4 s, thus after 1, 5, 9, 13, 17, 21 s. Furthermore, the maximum coil temperatures ($${T}_{7}{=T}_{\mathrm{max}})$$ reached with a certain time delay ($$\sim 2 s$$) after switching off the coils were recorded. Averaged over all five trials per single set, coils heated up to a maximum temperature of $$T_{\max }$$$$_{{{\text{room}}}}^{1s}$$ = 28.4 $$\pm 0.8^\circ \mathrm{C}$$ starting from room temperature ($${T}_{\mathrm{room}}$$), and of $$T_{\max }$$$$_{{{\text{body}}}}^{1s}$$ = 41.6 $$\pm 0.1^\circ \mathrm{C}$$ for starting at body temperature ($${T}_{\mathrm{body}}$$).

Based on these results, supplementary calculations were carried out to determine whether polymer-based encapsulation can achieve the desired and adequate shielding process.

The results from the heating assessment were used to calculate the required isolation width. Therefore, the admissible inner and outer wall temperatures had to be defined. With respect to safety–critical dimensioning, the maximum mean measured coil temperature $$T_{\max }$$$$_{{{\text{body}}}}^{1s}$$ = 41.6 $$\pm 0.1^\circ $$ C was rounded up to $${T}_{\text{inner}\, \text{wall}}=$$~$$42.0^\circ \mathrm{C}$$. The admissible outer wall temperature of the application device was set to $${T}_{\text{outer}\, \text{wall}}=$$~37.0 °C (to obviate tissue damage also in case of direct applicator wall contact).

During heat transfer through a wall, different phenomena, namely convection, radiation and conduction occur [24]. For a first approximation, the problem was simplified assuming direct contact between coil and encapsulation wall. Convection, radiation, and interfacial heat transfer phenomena were neglected for the time being. Furthermore, the polymer wall was assumed to be isotropic with respect to heat conduction properties. A current-carrying conductor, supplied by a continuously uniform energy, reaches a state of equilibrium at an elevated temperature, in which the heat transfer rate $\left( {\dot{Q}} \right)$ corresponds to the electrical power ${(P}_{\mathrm{el}})$ absorbed [Eq. 2]. Although the maximum temperature was measured directly after the coil had been deactivated, thus ${P}_{\mathrm{el}} \mathrm{being} \,0$ *W*, an enduring, continuous power of ${P}_{\mathrm{el}}= 3 \left[\mathrm{A}\right]*24 \left[\mathrm{V}\right]$ at 42.0 °C was assumed for the calculations. This approach was chosen to avoid under dimensioning of the heat shielding entity. Furthermore $, \dot{Q}$ was assumed to be steady. By using the equation for heat transfer through a cylindrical wall [Eq. 3] [25] derived from Fourier’s heat equation [Eq. 1] [25], a required wall thickness $\Delta r={r}_{2}-{r}_{1}$ was calculated, with $\dot{Q}$ representing the amount of heat transferred over a certain time, ${A}_{Q}$ being the cross-sectional area and *k* the material specific conductivity, with *k* $\in$ [0.1$\frac{W}{m*K}$; 0.5 $\frac{W}{m*K} ]$ for most polymers [25]. For the calculations, the upper limit value for safety reasons *k* = 0.5 $\frac{W}{m*K}$ was used. *L* is the cylinder length active for heat conduction with *L* = 15.0 mm,$\Delta T$ the temperature gradient over wall thickness with ${T}_{1}=42.0^\circ \mathrm{C}$ and${T}_{2}=37.0^\circ \mathrm{C}$, ${r}_{1}=\frac{6.4 \left[\mathrm{mm}\right]+0.3 \left[\mathrm{mm}\right]*6}{2}=4.1 \left[\mathrm{mm}\right] \mathrm{the}$ outer coil radius (including windings) and ${r}_{2}$ the outer encapsulation wall radius. Inserting all variables into the equations:

1 $$ \dot{Q} = \frac{Q}{\Delta t} = - k*A_{Q} *\frac{\Delta T}{{dr}} = - 2*k\pi rL\frac{\Delta T}{{dr}} $$

$$ \dot{Q}\mathop \smallint \limits_{{r_{1} }}^{{r_{2} }} \frac{1}{r}dr = - 2k\pi L\mathop \smallint \limits_{{T_{1} }}^{{T_{2} }} dT $$

$$ \dot{Q}*\left( {\ln \left( {r_{2} } \right) - \ln \left( {r_{1} } \right)} \right) = - 2k\pi L \left( {T_{2} - T_{1} } \right) $$

2 $$ \dot{Q} = P_{el} = U*I $$

3 $$ P_{el} = \dot{Q} = \frac{{2k\pi L \left( {T_{1} - T_{2} } \right)}}{{{\text{ln}}\left( {\frac{{r_{2} }}{{r_{1} }}} \right)}} $$

Solving for ${r}_{2}$ led to the following equation and a required shielding wall thickness of 0.013 mm.

$$ \begin{aligned} r_{2} = & r_{1} *e^{{\frac{{2k\pi l \left( {T_{1} - T_{2} } \right)}}{{P_{el} }}}} = 4.1 \left[ {{\text{mm}}} \right]* e^{{\frac{{2*0.5\left[ {\frac{W}{m*K}} \right]*\pi *15\left[ {{\text{mm}}} \right]*\left( {5.0 \left[ K \right]} \right)}}{24 \left[ V \right]*3 \left[ A \right]}}} \\ = & 4.1 \left[ {{\text{mm}}} \right]* e^{{3.27*10^{ - 3} }} = {4}.{113} \left[ {{\text{mm}}} \right] \\ \end{aligned} $$

$$ \Delta r = r_{2} - r_{1} = {4}.{113 }\left[ {{\text{mm}}\left] { \, {-}{ 4}.{1 }} \right[{\text{mm}}\left] { \, = \, 0.0{13 }} \right[{\text{mm}}} \right] $$

Thus, we assessed the approach is feasible for the integration into an endoscopic anastomosis tool, as the operation scenarios are very restricted. However, for more extensive operation durations, further safety measures would be required.

## Discussion

Developing magnetic devices for minimally invasive surgery was a significant advance and their potential for more complicated procedures, such as anastomosis formation, was already discovered by others [3]. Magnamosis™ and IMAS were the first to combine the compression implant approach with the technology of flexible endoscopy. However, with respect to endoscopic navigation and manipulation, the mutual attraction of magnetic components may increase the risk of positioning failure, and thus, of inadvertently pinching healthy structures, or may interfere with other surgical instruments, which are typically composed of alloys [3]. This is especially problematic, as due to the endoluminal approach, the access possibilities for the surgeon are very limited and readjustment by hand is not possible.

Therefore, the goal of this paper was to present an alternative approach, using electromagnets in an endoscopic platform to increase system controllability and flexibility by providing a distinctively triggerable and reusable release mechanism, to cope with the challenges of minimized access routes during minimally invasive interventions. Particular challenges were assumed with respect to the interaction of magnetic implant components with the electromagnets. These must provide sufficient power to overcome respective attraction forces and detach the implant. At the same time, the implant compression force must be sufficient to ensure a stable connection that can cope with the immediately postoperatively occurring forces.

For our end-to-end anastomosis, we achieved a mean separation force of ${F}_{{\text{electromagnet implant}}}^{\text{separation}}=$ 1.53 ± 0.3 N with two tissue layers of $\sim 2.9 mm$ thickness in total in the implant compression zone. This is comparable to the separation force of 1.48 N with a 95% confidence interval of [1.11 N-1.86 N] for the Magnamosis™ system, indicated by Wall et al. in their studies for porcine colorectal end-to-end anastomoses [10].

However, for an initial estimation of healing and necrosis behavior, compression pressure acting on tissue is more meaningful than force. Accordingly, this parameter was also determined for various implants reported in the literature and put into relation to our implant. Lambe et al. indicated excellent gastroenteric compression anastomoses quality in all animals of their survival study, for a range of compression pressures between 30 and 60 $\frac{N}{{\mathrm{cm}}^{2}}$ (0.3 and 0.6 $\frac{\mathrm{N}}{{\mathrm{mm}}^{2}}$), for 2 mm inter magnet distance, and a range of 1.0–3.5 $\frac{\mathrm{N}}{{\mathrm{mm}}^{2}}$ (which is 100–350 $\frac{\mathrm{N}}{{\mathrm{cm}}^{2}}$) for bilioenteric anastomoses (as well for 2 mm inter magnet separation) [6].

As the authors did not indicate the compression pressure for the Magnamosis™ implant, this was calculated for the colorectal end-to-end anastomosis. Neglecting any geometrical specifications with respect to the conically shaped compression zone, the pressure active surface area of the implant, which has an outer diameter of 23 mm and an inner diameter of 9.6 mm [5], is approximately.

$$ A_{\text{magnamosis implant}} = \left({ \frac{r_{\rm out}}{2} - \frac{r_{\rm in}}{2} } \right)^{2} *\pi = \left({ \frac{23}{2} - \frac{9.6}{2}}\right)^{2} *\pi = 141.03\;{\text{mm}}^{2} $$

This resulted in a pressure of

$$ p = \frac{{{\text{F}}_{{\text{magnamosis}\,\text{implant}}}^{\text {separation}} }}{{A_{{{\text{magnamosis}\, {\text{implant}}}}} }} = \frac{{1.48{\text{ N}}}}{{141.03{\text{ mm}}^{2} }} = 0.0105\;\frac{{\text{N}}}{{{\text{mm}}^{2} }} $$

Comparing the tissue compression of the various implants, which were specified as optimized healing conditions, to our implant with a pressure of ${P}_{\text{electromagnet implant}}$ = $0.0048 \frac{\mathrm{N}}{{\mathrm{mm}}^{2}}$, it becomes apparent that the magnitudes vary considerably for every implant and application. It was already postulated by Lambe et al. that the creation of magnetic compression anastomoses using permanent magnets for both gastroenteric and bilioenteric applications, demonstrate a high resilience to variations in magnetic force and pressure exerted [6].

The measurements performed with the Magnamosis™ implant for colorectal anastomoses are closest to our application.

The lower pressure realized by our implant suggests that necrosis processes may take longer, which is assumed to be less critical than a premature implant excretion. However, at this stage, no final conclusions can be drawn on how the pressure conditions of the implant affect the healing process of the tissue. Further investigations must be conducted to determine how the segmentation of the implant into areas with and without magnets generally affect the physiological processes.

Our research did not yet aim at demonstrating optimal healing conditions, but to answer the question whether the implant provides sufficient support and holding strength, to counteract the forces acting through peristalsis, or possible interactions with digestive products, which could eventually lead to an undesired slipping of the implant halves during the healing phase. This was confirmed by our measurements and the comparison to similar devices. Furthermore, in the second part of our experiments, we were able to demonstrate that the integrated electromagnets allowed to reproducibly apply forces at the endoscope tip, high enough, to detach the magnetic implant remotely.

Comparing the applicator head dimensions of our prototype to existing endoscopic platforms on the market, we were able to establish device dimensions similar to existing systems. The internal lumen diameter (until implant excretion) of our implant $(\sim$ 21.4 mm at narrowest point 18.6) is larger than that of the Harrison Rings (9.6 mm) [5], thus reducing the risk of fecal impaction [26]. The outer implant diameter of our current system is in the range of 34–35 mm in diagonal length, which is comparable to IMAS (27–35 mm) [27] and larger than the Magnamosis™ system (23 mm) [5]. This parameter influences the resulting anastomotic diameter after excretion of the implant. For stapling systems, the resulting anastomosis lumen diameters are in general smaller with 11–24 mm, often leading to stenosis of the intestinal lumen.

A further comparison can be drawn to other interventional endoscopic tools. Examples are the DDES (Direct Drive Endoscopic System (Boston Scientific, USA) with a diameter of ∅ = 22 mm, the Robotic system MASTER (NanyYang, Technological University, Singapore), also with a diameter of ∅ = 22 mm, the EndoSamurai (Olympus, Japan) with a diameter of ∅ = 18 mm and the Incisionless operating platform (USGI, USA) with a diameter of ∅ = 18 mm [28]. Most of these systems are specifically dedicated to the application through the pharynx. Due to the anatomical restrictions, the outer diameter here is limited to less than 20 mm [28]. However, we assume that with respect to the cartilage-free wall structure and the greater flexibility of colon tissue, larger device dimensions are admissible for a colonoscopic system. This assumption is supported by related endoscopic tools, dedicated to the use within the colon, such as the Meshworm, which was developed by Bernth et al. [29]. It has an outer diameter of 31 mm when uncompressed, and 35 mm when compressed, which is comparable to our device dimensions. However, comparing both systems, we assume the anastomotic device’s restricted degree of flexibility to be more critical than the diameter dimensions. In contrast to the Meshworm, which consists mainly of an elastic net, our anastomosis systems largely comprises rigid parts. This has to be critically reflected with respect to the complex colon topology, considering navigation around the curvatures at the sigma and the flexures.

Finally, heat emission resulting from the electromagnet operation was investigated to identify potential patient hazards. If heat is applied to tissue, thermal effects occur in cells that are exposed to temperatures below or above a thermo-neutral zone *T* $\in$ [35 °C; 41.5 °C] [30]. Beyond these thresholds, irreversible thermal effects occur, which may lead to permanent cell damage. If cellular structures are heated to temperatures between 41.5 and 49.0 °C this might lead to invisible devitalization. The affected tissue can subsequently disintegrate, leading to life threatening complications, such as delayed perforations and intracorporeal bleeding [30]. While exposure time in this respect is of great importance, immediate devitalization must be expected, if temperature rises even further above 49.0 °C [30].

Thus, heat emission resulting from the electromagnet operation was investigated. The experiments revealed that the maximum temperatures reached critical levels beyond the thermo-neutral zone of 41.5 °C for the pulsed $$T_{\max }$$$$_{{{\text{body}}}}^{1 s} = 41.6 \pm 0.1\;^\circ {\text{C }}$$.

A polymeric isolation of the hot elements can support preclusion of tissue damage. However, due to the tight size restrictions for endoscopic instruments, the electromagnet encapsulation must be limited. Based on the experiments conducted and accounting for heat transfer effects, additional preventive design specifications were derived.

Thus, for the further development a polymeric encapsulation was considered as a shielding safety measure. Heat transfer calculations showed that a wall width of 0.013 mm would already be feasible. Based on these findings, it was derived that for an anastomosis device, which uses an electromagnetic actuation, critical thresholds for the isolating wall are determined rather by stability considerations and accuracy limitations of the manufacturing process, than by heat shielding requirements. Thus, the integration of electromagnets into endoscopic devices seems feasible, allowing for a safe and force-saving operation with respect to heating effects.

Electromagnet encapsulation is needed not only to shield heat, but also to insulate all active components. The majority of our organs and movements is controlled by electrical impulses originating from the brain. Extraneous current flowing through the body can lead to muscle cramping and may affect organ functions, if it is significantly greater than the body’s own. An AC voltage of 50 V and a DC voltage of 120 V can have life-threatening consequences. With approximately 24 V, the required DC voltage for our system is far below this critical threshold. [31]

### Conclusion

Based on our studies conducted, we conclude that electromagnets bear high potential for the application in complex endoscopic platforms and may help to overcome significant problems of modern endoscopic surgery. They allow for the application of high forces remotely at the endoscope tip, without requiring stiff endoscope shafts to transfer forces. Furthermore, they enable high system controllability and flexibility by providing a distinctively triggerable and reusable release mechanism, to cope with the challenges of minimized access routes during minimally invasive interventions.

Further research must focus on fine-tuning electromagnet and implant interaction. Parameters to adapt this interdependency include coil dimensions and the number of windings, current, wire thickness, strength and shape of the permanent magnet, implant and electromagnet polymeric encapsulations (material/wall width), as well as coil core material. Further investigations must be performed to assess how long establishment of a neat, continuous and healed anastomosis formation takes within an organism and the time until the implant is expelled from the colon.

## Footnotes

1 Magnet shop Neodym Magnets 5 × 5x5 mm NdFeB N52, https://www.magnethandel.de/neodym-magnete-5-5-5-mm-n52#tab-description. last accessed July 6, 2022.

2 Re-In Retail International GmbH 803588 Screw assortment 3000 parts, https://www.voelkner.de/products/74881/803588-Kombi-Schrauben-Sortiment-3000-Teile.html; Product sheet: https://asset.re-in.de/add/160267/c1/-/de/000803588IN01/IN_803588-Kombi-Schrauben-Sortiment-3000-Teile.pdf; last accessed July 6, 2022.
